# Beyond prediction intervals in meta-analysis: reporting the expected proportion of comparable studies with clinically relevant benefit or harm

**DOI:** 10.1186/s12874-025-02733-9

**Published:** 2025-12-07

**Authors:** W. Siemens, M. Borenstein, T. Evrenoglou, J.J. Meerpohl, G. Schwarzer

**Affiliations:** 1 Institute for Evidence in Medicine, Medical Center - University of Freiburg / Medical Faculty - University of Freiburg, Freiburg, Germany; 2 Biostat, Inc, New York, NY USA; 3 Institute of Medical Biometry and Statistics, Faculty of Medicine and Medical Center - University of Freiburg, Freiburg, Germany; 4 Cochrane Germany, Cochrane Germany Foundation, Freiburg, Germany

**Keywords:** Heterogeneity, Meta-analysis, Random-effects, Prediction interval, Proportion, Probability

## Abstract

**Background:**

In a meta-analysis where the effect size varies substantially between studies it is important to report the extent of the variation. Critically, we want to know if the treatment is always helpful or sometimes harmful. The statistic that addresses this is the prediction interval (PI), which gives the range of true effects for all studies comparable to those in the meta-analysis.

**Methods:**

In addition to the PI’s upper and lower limits, we propose to report the expected proportion of comparable studies that are expected to have an effect in a given range. If we define for example thresholds corresponding to minimal clinically important benefit and harm, we can report the expected proportion of comparable studies where the true effect is expected to exceed these thresholds.

**Results:**

We apply our approach to two Cochrane Reviews assessing a dichotomous and a continuous outcome: caesarean section and health-related quality of life. This article shows how to plot the distribution of true study effects highlighting the expected proportion of comparable studies where the true effect is clinically beneficial or harmful. We also offer suggestions for how to report this information in scientific articles.

**Conclusion:**

In addition to PIs, reporting the expected proportion of comparable studies with relevant benefit or harm as supplementary information could help physicians and other decision-makers to understand the potential utility of an intervention. However, these metrics must be interpreted with caution because the estimate of the between‑study heterogeneity ${\tau }^{2}$ may be imprecise when data are limited.

**Supplementary Information:**

The online version contains supplementary material available at 10.1186/s12874-025-02733-9.

## Background

Heterogeneity, or the variation in effect sizes across studies, poses a significant challenge in interpreting findings from random-effects meta-analyses. This issue is especially pronounced in fields where intervention effects vary widely across populations [1].

The confidence interval (CI), sometimes misunderstood as a measure of heterogeneity, is actually an index of precision. It tells us how precisely we have estimated the mean effect size, but says nothing about the dispersion in study effects. Another index often interpreted as telling us how much the effect size varies is *I*^*2*^. However, *I*^*2*^ only tells us what *proportion* of the observed variance reflects variance in true effects rather than sampling error. It does not provide any information on the variation of study effects [2–5]. *I*^*2*^ does not indicate, for example, if some studies overlap with the null effect or a clinically meaningful threshold. Critically, neither the CI nor *I*^*2*^ convey information on the range of study effects in meta-analysis [1, 5–7].

The statistics $\widehat{\tau }$ and ${\widehat{\tau }}^{2}$ estimate the standard deviation and variance of the true study effects in a meta-analysis. However, while $\tau$ may tell us that the effect varies over a certain span, it does not tell us if the effect varies from a small benefit to a large benefit, or from harmful to beneficial. Additionally, these statistics are sometimes reported in log units, which are not intuitive. They do not provide the information the reader actually needs, which is the range of true study effects [1, 5].

The one statistic that does tell us the range of true study effects in a random-effects meta-analysis is the prediction interval (PI). For example, if the PI ranges from a risk ratio (RR) of 0.25 to 1.25, we know that in 95% of studies comparable to those in the meta-analysis, the true effect size will fall in this range, meaning a decrease in risk by a factor of 4 to an increase in risk by a factor of 1.25 compared to the control [1, 5–8].

In addition to the lower and upper limits of the PI, we propose to report the expected proportion of comparable studies that will have true effects within a specified range. By defining thresholds for the minimal clinically important benefit and harm, we can, for instance, conclude that a treatment is likely to be beneficial in 40% and harmful in 10% of studies comparable to those in the meta-analysis.

The concept of estimating the proportions (or probabilities) of future comparable studies indicating benefit and harm has been previously discussed [5] and further elaborated [9–11]. First, our approach differs from the previous methods because we use the latest recommendation to calculate the PI in line with Cochrane [8] (t-distribution, degrees of freedom: *K* studies −1, incorporation of the estimates ${\widehat{\tau }}^{2}$ and the variance of the pooled estimate in the random-effects meta-analysis ${SE\left({\widehat{\mu }}_{RE}\right)}^{2}$) [8, 12] instead of using the standard normal distribution. Second, we implemented our approach in the R package **meta** [13] enabling easy calculation and visualization of the results. We acknowledge that Mathur & VanderWeele extended their approach by nonparametric calibrated methods [10], and cluster-bootstrapping methods [14].

This article demonstrates how to visualize the distribution of true study effects and how to illustrate the expected proportion of studies where the true effects are clinically beneficial or harmful, given clinically relevant thresholds. It also provides guidance on how to effectively report this information in research articles.

## Methods

### Use cases: meta-analyses from two Cochrane reviews

To elaborate the visualization and calculation of the expected proportion of comparable studies indicating clinically important benefit or harm, we chose meta-analyses from two Cochrane Reviews [15, 16]. These use cases illustrate the topic by presenting effect measures for dichotomous and continuous outcomes, together with different levels of heterogeneity, each with potentially different clinical implications.

#### Use case 1: caesarean section

The first use case is a meta-analysis of 16 studies from the Cochrane Review by Sandall et al. [16] evaluating the effect of midwife continuity of care models compared to other models of care on caesarean birth (Table 1). Midwifery continuity of care models involve the same midwife or a consistent team providing care throughout pregnancy, childbirth, and the early parenting phase. The midwives work alongside obstetric and specialized medical teams as needed aiming to increase spontaneous vaginal birth and to reduce caesarean sections.

**Table 1:** Characteristics of included Cochrane reviews extracted from the summary of findings tables

**Use cases**	**Participants and setting**	**Intervention**	**Control**	**Outcomes analyzed in this article, effect measure, MID**
Use case 1: Sandall et al. (2024) [16]	-Childbearing women and their infants -Hospital and community-based environments where midwife continuity of care and other care models are implemented for childbearing women and their infants	-Midwife continuity of care models	-Other models of care	-Caesarean birth, RR, MID: 0.8 and 1.25
Use case 2: Molloy et al. (2024) [15]	-Adults with heart failure -Centre-based, home-based, and hybrid settings	-Exercise-based cardiac rehabilitation	-Usual care (with other active interventions)	-HRQoL, MD, MID: ±5 points on MLWHF questionnaire

*HRQoL* Health-related quality of life, *MID* Minimal important difference, *MD* Mean difference, *MLWHF* Minnesota Living With Heart Failure questionnaire (range 0–105; large values: bad quality of life), *RR* Risk Ratio

We chose this example because midwifery is an established medical field, and caesarian section is considered a relevant outcome for women – as long as the caesarean section is not performed by the conscious choice of the woman. Moreover, a dichotomous outcome with RR as the effect size index is very common. As a rough guide for defining a clinically relevant effect, we derived thresholds based on Guyatt et al. [17] and chose an RR of 0.8 and 1.25 for relevant benefit and harm, respectively. We recognize that these thresholds are somewhat arbitrary and are based on relative, not absolute effects. Furthermore, while these thresholds are symmetric (in log units) around an RR of 1, we could also use asymmetric thresholds.

#### Use case 2: health-related quality of life

The second use case is a meta-analysis of 22 studies from the Cochrane Review by Molloy et al. [15] assessing the effect of exercise-based cardiac rehabilitation compared to usual care (with other active interventions) on health-related quality of life measured by the Minnesota Living With Heart Failure (MLWHF) questionnaire. The questionnaire scores can range from 0 to 105, with high scores indicating poor quality of life (Table 1). Cardiac rehabilitation aims at supporting individuals in recovering from heart conditions such as heart failure. These programs often include exercise training and may offer guidance on lifestyle changes, risk factor management, as well as counseling and psychological support.

We selected this example because cardiology is an established medical field and health-related quality of life is a relevant outcome for patients suffering from heart failure. Additionally, when assessing quality of life, using a continuous outcome with the mean difference (MD) as the effect measure is common. Based on the Cochrane Review and a methodological study examining the MLWHF questionnaire's responsiveness [15, 18], we considered an overall difference of ±5 points MLWHF a clinically relevant benefit and harm.

### Random-effects meta-analysis model

For comparing two treatments, let $K$ be the number of studies and ${y}_{i}$ ($i=1, 2,\dots ,K$) be the observed effect size in study $i$. ${\theta }_{i}$ is the true study-specific treatment effect and ${\sigma }_{i}^{2}$ the true variance of ${y}_{i}$ from study $i$. Then, the standard random-effects Inverse Variance (IV) model can be parametrized using the following hierarchical expression [8, 19],

$${y}_{i}\sim N\left({\theta }_{i},{\sigma }_{i}^{2}\right)$$

$${\theta }_{i}\sim N({\mu }_{RE},{\tau }^{2})$$

where the study-specific true treatment effects ${\theta }_{i}$ vary around a common mean denoted as ${\mu }_{RE}$ with variance equal to ${\tau }^{2}$, which represent the heterogeneity across studies. The parameters of interest in this model are the parameters ${\mu }_{RE}$ and ${\tau }^{2}$. In this manuscript we fit the IV model and we get the estimates ${\widehat{\mu }}_{RE}$ and ${\widehat{\tau }}^{2}$ within the frequentist framework. In order to exactly re-calculate the use cases, we estimate ${\mu }_{RE}$ using the weighted least squares approach [20, 21] and ${\tau }^{2}$ using the DerSimonian-Laird (DL) estimator [19]. Alternative options to estimate ${\tau }^{2}$ are also possible (e.g., the often recommended restricted maximum likelihood method) and have been discussed elsewhere [12].

### Calculation of the PI

The PI is a measure of heterogeneity in random-effects meta-analyses. It addresses the range of true effects across all studies comparable to those included in the meta-analysis. PIs are calculated using a *t*-distribution and provide insight into the distribution of effects across studies by incorporating an estimate for the between-study variance ${\tau }^{2}$ [1, 5, 6]. In contrast to other approaches that rely on the standard normal distribution [9] or “calibrated” estimates to address overdispersion allowing for non-normal true effect distributions and including bootstrapping for inference [10], the 95% PI formula used in this article is based on the work of Veroniki et al. [12] in which the 95% PI is defined as:

1 $$PI = {\widehat{\mu }}_{RE}\pm {t}_{K-1, 0.975} \sqrt{\left\{{\widehat{\tau }}^{2}+ {SE\left({\widehat{\mu }}_{RE}\right)}^{2}\right\}}$$

In the random-effects model, the pooled estimate is represented as ${\widehat{\mu }}_{RE}$. Here, ${t}_{K-1, 0.975}$ denotes the 97.5% quantile of the t-distribution with *K-1* degrees of freedom resulting in a 95% PI, where *K* is the number of studies. ${\widehat{\tau }}^{2}$ represents the estimated between-study variance, and ${SE\left({\widehat{\mu }}_{RE}\right)}^{2}$ is the estimated variance of the pooled estimate in the random-effects meta-analysis. Equation (1) gives us a lower and an upper limit of the PI in the units of the meta-analysis, e.g. in use case 1 for a dichotomous outcome the log RR or in use case 2 for a continuous outcome the MD. Please note that Higgins et al. used *K-2* degrees of freedom [6], whereas we adopt the more recent formula of Veroniki et al. with *K-1* degrees of freedom [12]. This choice yields the same number of degrees of freedom for both CIs and PIs, so that the CI and PI are identical when the estimator of the between-study variance ${\widehat{\tau }}^{2}$ is zero.

### Deriving probabilistic metrics based on the PI

Let *CID1* and *CID2* be the (transformed) lower and upper clinically relevant thresholds, i.e., CID1 = log(0.8) and CID2 = log(1.25) in use case 1, and CID1 = −5 and CID2 = 5 in use case 2. We calculate expected proportions of comparable studies below the lower or above the upper clinically relevant threshold by computing the area under the t-distribution underlying the PI.

2 $$P\left(below\right)={\int }_{-\infty }^{CID1}{f}_{df}\left(x\right)\text{d}x$$

3 $$P\left(above\right)={\int }_{CID2}^{+\infty }{f}_{df}\left(x\right)\text{d}x$$

The area between the lower and upper threshold corresponds to the expected proportion of comparable studies having clinically irrelevant effects:

4 $$P\left(irrelevant\right)=1-P\left(below\right)-P\left(above\right)$$

These equations are based on the integration of the density function ${f}_{df}\left(x\right)$ of a t-distribution with degrees of freedom *df = K-1*.

### Visualization of expected proportions for clinically relevant benefit and harm

This procedure has been implemented in the R package **meta**, function *cidprop()*, and can be visualized using the function *plot.cidprop()* [13]. Alternatively, the freely available software CMA Prediction Intervals may be used to create such plots and may be download at www.Meta-Analysis.com.

## Results

We re-calculated both meta-analyses [15, 16] using the following settings from Cochrane’s software RevMan [22]: The inverse variance method was used as weighting method, the DL estimator was selected for estimating the between-study variance ${\tau }^{2}$, and z-quantiles were used for calculating CIs which are based on a standard normal distribution (Wald-type test). We used software R version 4.4.1 [23], package **meta** [13] for all re-calculations.

**Fig. 1:**
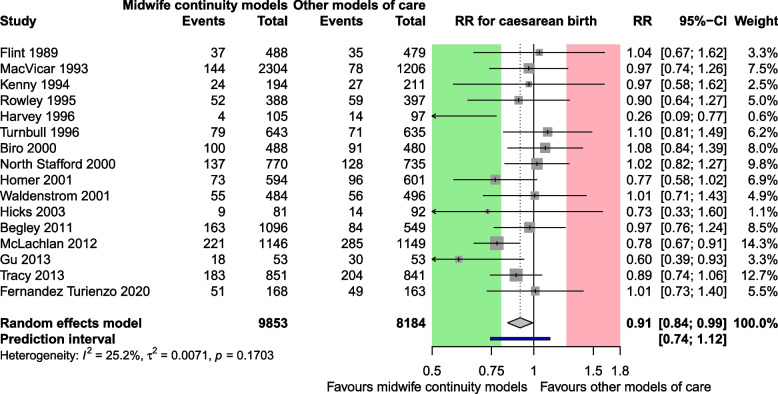
Random-effects meta-analysis of use case 1: caesarean section. CI: confidence interval; MLWHF: Minnesota Living With Heart Failure questionnaire (range 0–105; large values: bad quality of life); PI: prediction interval; RR: Risk Ratio; SD: standard deviation. Cutoffs for clinically relevant benefit and harm: 0.8 and 1.25

**Fig. 2:**
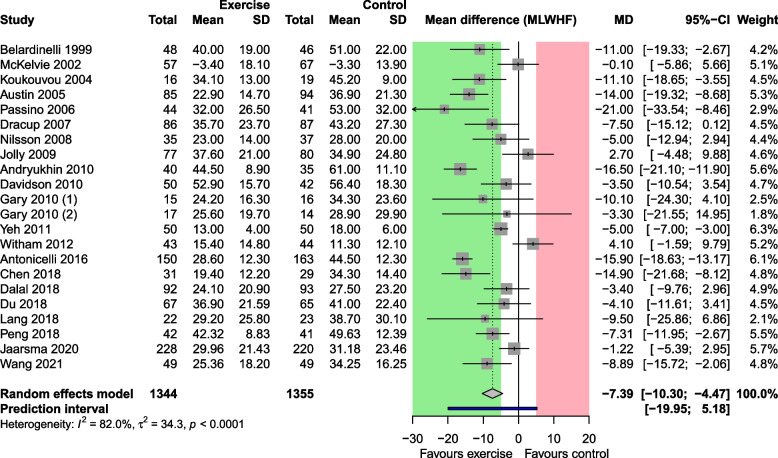
Random-effects meta-analysis of use case 2: health-related quality of life. CI: confidence interval; MD: mean difference; PI: prediction interval; SD: standard deviation. Cutoffs for clinically relevant benefit and harm: −5 and 5

Figures 1 and 2 show the random-effects meta-analyses of use case 1 (caesarean section) and use case 2 (health-related quality of life), respectively. The areas of clinically important benefit and harm are highlighted in green and red.

### Summary of meta-analysis results including PI

#### Use case 1: caesarean section

Midwife continuity care models reduced the risk of caesarean sections on average by a factor of 0.91 (95% CI 0.84 to 0.99). Critically, the CI addresses only the mean effect size, and fully lies within the region of clinically irrelevant effects, i.e., within the interval RR 0.80 to RR 1.25. By contrast, the PI illustrates the dispersion of the study effects. It tells us that in 95% of all studies comparable to those in the meta-analysis, the true RR is likely to fall in the range of 0.74 to 1.12. Thus, the PI crosses the threshold for relevant benefit of RR 0.80 and includes the null effect. However, it excludes the threshold for relevant harm of RR 1.25 (Fig. 1).

#### Use case 2: health-related quality of life

Exercise-based cardiac rehabilitation improved the health-related quality of life on average by −7.39 points (95% CI −10.30 to −4.47) on the MLWHF questionnaire, and this mean is larger than the relevant benefit threshold of −5 points. Again, the CI addresses only the mean effect size, and tells us that the mean effect is almost certainly clinically important. By contrast, the PI indicates that in 95% of all studies comparable to those in the meta-analysis, the true MD is likely to fall in the range of −19.95 to 5.18. The PI crosses the threshold for relevant benefit of MD −5, and the null effect. It also crosses the threshold for relevant harm of MD +5 points but just barely (Fig 2).

### Expected proportion of studies indicating relevant benefit or harm

The PI gives helpful information in both use cases on the range of true effect sizes in studies comparable to those in the meta-analysis. However, the PI does not provide information on the expected proportion of comparable studies where the effect will be clinically beneficial, harmful, or neutral. This is addressed by the approach presented in this article.

#### Use case 1: caesarean section

**Fig. 3:**
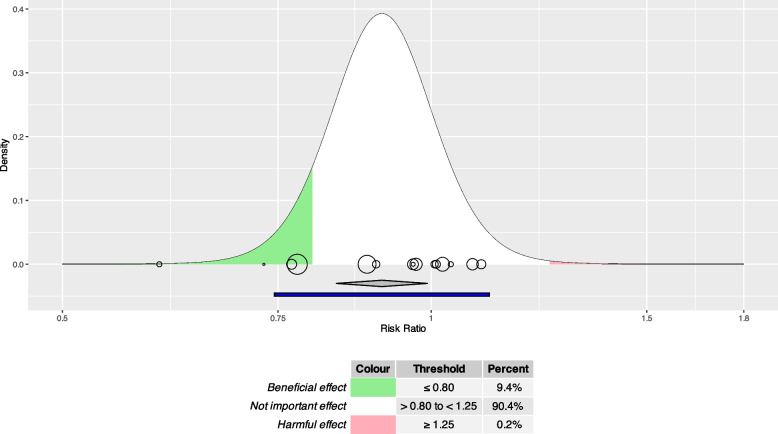
Expected proportion plot for use case 1: caesarean section. RR: Risk Ratio. Cutoffs for clinically relevant benefit and harm: 0.8 and 1.25. Green and red area: Midwife continuity care models are expected to be clinically helpful (RR ≤ 0.80, green area) in 9.4% and to be clinically harmful (RR ≥ 1.25, red area) in 0.2% of studies comparable to those in the meta-analysis. Circles: size represents weight and position represents effect of each study included in meta-analysis. Grey diamond: pooled effect and 95% confidence interval; 95% prediction interval shown in blue under the grey diamond

Figure 3 indicates that midwife continuity care models are expected to be clinically helpful (RR ≤ 0.8) in about 9.4% and clinically harmful (RR ≥ 1.25) in about 0.2% of studies comparable to those in the meta-analysis. Following equation (4), the expected proportion of comparable studies with irrelevant effects (0.8 < RR < 1.25) is estimated as 100% - 9.4% - 0.2% = 90.4%.

#### Use case 2: health-related quality of life

**Fig. 4:**
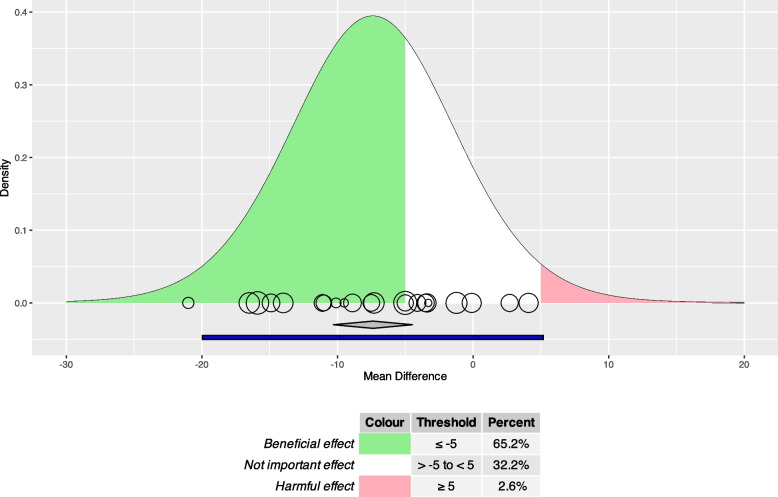
Expected proportion plot for use case 2: health-related quality of life. MD: mean difference; MD of the Minnesota Living With Heart Failure questionnaire (range 0–105; large values: bad quality of life). Green and red area: Exercise-based cardiac rehabilitation is expected to be clinically helpful (MD ≤ −5; green area) in 65.2% and to be clinically harmful (MD ≥ 5, red area) in 2.6% of studies comparable to those in the meta-analysis. Circles: size represents weight and position represents effect of each study included in meta-analysis. Cutoffs for relevant benefit and harm: −5 and 5. Grey diamond: pooled effect and 95% confidence interval; 95% prediction interval shown in blue under the grey diamond

Figure 4 shows that exercise-based cardiac rehabilitation is expected to be clinically helpful (MD ≤ −5) in about 65.2% and clinically harmful (MD ≥ 5) in about 2.6% of studies comparable to those in the meta-analysis. This leaves an expected proportion of about 32.2% (100% - 65.2% - 2.6%) of comparable studies to result in irrelevant effects.

### Suggestions for wording

Since researchers often find it challenging to convey a numeric result of the PI and the expected proportion of clinical benefit and harm effectively in narrative form, we propose example language to ensure accurate communication (see Box).

**Table:** 

**Box: Suggested wording to communicate the results of the prediction interval (PI) and the expected proportion of studies showing clinical benefit or harm:**
**Use case 1: caesarean section**
a) In 95% of all studies comparable to those in the meta-analysis, the true RR is likely to fall in the range of 0.74 to 1.12. b) Midwife continuity care models are expected to be clinically helpful (RR ≤ 0.80) in about 9.4% and to be clinically harmful (RR ≥ 1.25) in about 0.2% of studies comparable to those in the meta-analysis. We expect about 90.4% of comparable studies would fall in the range of trivial effects (RR >0.8 to < 1.25).
**Use case 2: health-related quality of life**
a) In 95% of all studies comparable to those in the meta-analysis, the true MD is likely to fall in the range of −19.98 to 5.21. b) Exercise-based cardiac rehabilitation is expected to be clinically helpful (MD ≤ −5) in about 65.2% and to be clinically harmful (MD ≥ 5) in about 2.6% of studies comparable to those in the meta-analysis. We expect about 32.2% of comparable studies would fall in the range of trivial effects (MD > −5 to < 5).

*MD* Mean difference, *MLWHF* Minnesota Living With Heart Failure questionnaire, *RR* Risk ratio

## Discussion

In any meta-analysis where the effect size varies between studies it is imperative to report the PI, which tells us the range of true effects we expect to see in 95% of studies comparable to those in the meta-analysis. In this article we suggest to additionally report the expected proportion of comparable studies indicating clinical benefit or harm.

Although the PI has been introduced and endorsed many years ago [1, 5–7], and is supported by Cochrane [24], PIs have yet to see widespread adoption among researchers [25–28]. However, the integration of PIs in Cochrane’s software RevMan [22], along with methodological publications on PIs for both pairwise and network meta-analysis [26, 27, 29–34], as well as educational resources on PIs [1–5] are expected to increase the reporting of PIs.

Interestingly, PIs were not included in the latest Grading of Recommendations Assessment, Development, and Evaluation (GRADE) guidance on addressing inconsistency [35], although recent methodological work has explored their potential role in evaluating both imprecision and inconsistency together [36]. Considering the role of PIs in the GRADE framework to assess inconsistency when evaluating the certainty of the evidence is a promising direction of future research [17, 36–38].

We used the t-distribution to estimate the PI and to calculate the expected proportions of comparable studies for relevant benefit and harm, as recommended by Cochrane [8, 12]. For comparison, we also applied the “calibrated” and “parametric” (based on the standard normal distribution) approach suggested by Mathur et al. [9, 10] using R package MetaUtility [11] with 1000 bootstrap iterations, which yielded slightly different results (see section Data availability). However, we argue that consistency is crucial in this context, and therefore, if the PI is calculated and reported based on the t-distribution, it is logical to use the same statistical method for calculating the expected proportions of studies to maintain methodological coherence. We acknowledge that Mathur & VanderWeele extended their approach by cluster-bootstrapping methods [14].

### Methodological limitations

The reliable estimation of the PI and the expected proportion of studies showing a clinically relevant benefit or harm depends not only on the number of studies, but also on study size and the magnitude of variation of true study effects. Although use case 1 includes 16 studies, the 95% CI of ${\widehat{\tau }}^{2}$ is not calculable. Use case 2 includes 22 studies (${\widehat{\tau }}^{2}$: 34.3 [95% CI: 12.5 to 77.1]). In order to assess the uncertainty of these findings we implemented the lower and upper limits of ${\widehat{\tau }}^{2}$ to calculate the PI and received PIs of −15.03 to 0.29 and −26.21 to 11.31, respectively (using ${\widehat{\tau }}^{2}$: −19.95 to 5.18, see also Figure 2). For the expected proportion of studies, we estimate 73.6% for beneficial effects and 0.1% for harmful effects with the lower limit of ${\widehat{\tau }}^{2}$ and 60.6% for beneficial effects and 9.1% for harmful effects with the upper limit of ${\widehat{\tau }}^{2}$ (using ${\widehat{\tau }}^{2}$: beneficial effects: 65.2%, harmful effects: 2.6% effects, see also Fig. 4). This illustrates that the estimation of ${\tau }^{2}$ can become imprecise or even not feasible, which leads to uncertainty in the estimation of the PI and the expected proportion of studies. Consequently, these results should be interpreted with caution. However, the choice of the ${\tau }^{2}$ estimation method had no relevant impact on the PI and the expected proportion of studies when using the restricted maximum likelihood or the Paule-Mandel method (see sensitivity analyses in Supplementary Material) [39].

For meta-analyses addressing narrow clinical questions, involving relatively large studies with minimal methodological variation, at least 5 studies may be sufficient for robust estimation [8]. In contrast, when meta-analyses address broader questions, include smaller studies, or exhibit relevant methodological diversity, the imprecise estimation of ${\tau }^{2}$ is a limitation that needs to be acknowledged. In such contexts, acceptable estimation of the PI and the expected proportion of studies showing a clinically relevant benefit or harm likely requires preferably at least 10 studies.

Furthermore, while the PI provides an interval where roughly 95% of the true effects of comparable studies can be expected, it does not identify the factors contributing to heterogeneity across studies. For systematic review users, interpreting wide PIs without understanding the sources of variability can be challenging, as wide PIs may obscure whether the variability arises from clinical differences, methodological variations, or random sampling error. Identifying the sources of heterogeneity and communicating them adequately is one of the main tasks when conducting a systematic review [24].

A key assumption of the random-effects model is that the true study effects follow a normal distribution. However, testing for normality is not likely to be informative in this context. Instead, Borenstein [1] proposes a pragmatic approach to evaluate the plausibility of the PI: (a) if the PI indicates the treatment is consistently beneficial, there should be no studies showing clear evidence of harm; (b) if the PI suggests the treatment may be beneficial in some cases and harmful in others, studies should reflect evidence of both effects; and (c) if the PI indicates the treatment is consistently harmful, no studies should show clear evidence of benefit [1]. This pragmatic approach assumes that there is no publication bias.

Although we provided a rationale for the chosen thresholds [15, 17, 18], we recognize that the thresholds for determining the expected proportion of clinically relevant benefit and harm were chosen for didactic reasons. Should alternative thresholds be justified, the meta-analysis can be repeated to assess the robustness of the findings through sensitivity analyses. Ideally, review authors should use patient-centered, anchor-based thresholds that are validated for the specific population and instrument and incorporate stakeholder perspectives (e.g., patient groups/representatives, clinicians) [40]. For use case 1, the thresholds are defined by a relative effect of risk ratios (RR ≤ 0.8 or RR ≥ 1.25). They are not based on an absolute effect such as a risk difference, which the GRADE approach recommends using for defining decision thresholds [41]. As no clinical thresholds for absolute effects were used for caesarean birth in [16], we pragmatically derived these thresholds based on Guyatt et al. [17]. Nevertheless, since relative effects are typically more consistent across subgroups than absolute effects [42], the GRADE approach for rating down due to inconsistency applies to relative effects in binary outcomes [35]. In summary, threshold decisions should be taken before conducting the meta-analysis and should stem from a thorough review of the methodological and clinical literature, and from consensus within the interdisciplinary systematic review author team, ideally incorporating the patients’ perspective.

## Conclusion

The PI and the expected proportion for benefit and harm can be calculated with the R package **meta** via the function *cidprop() and plot.cidprop()* and with CMA Prediction Intervals. If the criteria for a reliable estimation of the PI are met, the PI provides the 95% range of true effect sizes for studies comparable to those in the meta-analysis. The PI and, as supplementary information, the expected proportion of comparable studies indicating relevant benefit or harm could substantially assist physicians and other decision-makers, such as guideline developers, in understanding treatment effects and their variability. Nonetheless, these metrics should be used cautiously, as the estimate for the between‑study heterogeneity ${\tau }^{2}$ can be imprecise when the data are sparse.

## Supplementary Information

Supplementary Material

## Data Availability

The data and R code to reproduce all calculations and figures of this article is freely available on GitHub: https://github.com/guido-s/cidprop-manuscript.
