# Supplementary material for: Beyond prediction intervals in meta-analysis: reporting the expected proportion of comparable studies with clinically relevant benefit or harm
Source: BMC Med Res Methodol. 2025 Dec 7;25:275. doi: 10.1186/s12874-025-02733-9 (PMC12696960; doi:10.1186/s12874-025-02733-9)
Supplement: Supplementary file 1 — Supplementary Material [file 12874_2025_2733_MOESM1_ESM.docx]

**Appendix**

# Sensitivity analyses:

## Use case 1:

### 1. Using 95% confidence limits of $\hat{\tau}^{2}$ to calculate prediction interval and expected proportions of studies

| ${\hat{\boldsymbol{\tau}}}^{\boldsymbol{2}}$ *** (95% CI)** | 0.0071 (NA; NA) |
| --- | --- |
| **95% prediction interval using lower limit** | NA; NA |
| **95% prediction interval using point estimate** | 0.74 to 1.12 |
| **95% prediction interval using upper limit** | NA; NA |
| **Expected proportion of studies using lower limit** | NA |
| **Expected proportion of studies using point estimate** | Beneficial^#^ effect: 9.4%  Not important^#^ effect: 90.3%  Harmful effect^#^: 0.2% |
| **Expected proportion of studies using upper limit** | NA |

* DerSimonian-Laird estimator for $\hat{\tau}^{2}$ representing the estimated between-study variance; # beneficial effect RR ≤ 0.80, not important effect RR > 0.80 to < 1.25, harmful effect RR ≥ 1.25

NA: not applicable, RR: risk ratio

### 2. Impact of other $\hat{\tau}^{2}$ estimates on prediction interval and expected proportions of studies

|  | **DerSimonian-Laird estimator for** ${\hat{\boldsymbol{\tau}}}^{\boldsymbol{2}}$ |
| --- | --- |
| **95% prediction interval** | 0.74 to 1.12 |
| **Expected proportion of studies** | Beneficial^#^ effect: 9.4%  Not important^#^ effect: 90.3%  Harmful effect^#^: 0.2% |
|  | **REML estimator for** ${\hat{\boldsymbol{\tau}}}^{\boldsymbol{2}}$ |
| **95% prediction interval** | 0.77 to 1.07 |
| **Expected proportion of studies** | Beneficial^#^ effect: 5.8%  Not important^#^ effect: 94.1%  Harmful effect^#^: <0.1% |
|  | **Paule-Mandel estimator for** ${\hat{\boldsymbol{\tau}}}^{\boldsymbol{2}}$ |
| **95% prediction interval** | 0.73 to 1.13 |
| **Expected proportion of studies** | Beneficial^#^ effect: 10.9%  Not important^#^ effect: 88.7%  Harmful effect^#^: 0.4% |

$\hat{\tau}^{2}$ represents the estimated between-study variance

# beneficial effect RR ≤ 0.80, not important effect RR > 0.80 to < 1.25, harmful effect RR ≥ 1.25

REML: Restricted maximum-likelihood estimator, RR: risk ratio

## Use case 2:

### 1. Using 95% confidence limits of $\hat{\tau}^{2}$ to calculate prediction interval and expected proportions of studies

| ${\hat{\boldsymbol{\tau}}}^{\boldsymbol{2}}$ *** (95% CI)** | 34.3 (12.5 to 77.1) |
| --- | --- |
| **95% prediction interval using lower limit** | -15.03 to 0.29 |
| **95% prediction interval using point estimate** | -19.95 to 5.18 |
| **95% prediction interval using upper limit** | -26.21 to 11.31 |
| **Expected proportion of studies using lower limit** | Beneficial^#^ effect: 73.6%  Not important^#^ effect: 26.2%  Harmful effect^#^: 0.1% |
| **Expected proportion of studies using point estimate** | Beneficial^#^ effect: 65.2%  Not important^#^ effect: 32.2%  Harmful effect^#^: 2.6% |
| **Expected proportion of studies using upper limit** | Beneficial^#^ effect: 60.6%  Not important^#^ effect: 30.3%  Harmful effect^#^: 9.1% |

* DerSimonian-Laird estimator for $\hat{\tau}^{2}$ representing the estimated between-study variance; # beneficial effect MD ≤ -5, not important effect MD > -5 to < 5, MD ≥ 5

MD: mean difference; NA: not applicable

### 2. Impact of other $\hat{\tau}^{2}$ estimates on prediction interval and expected proportions of studies

|  | **DerSimonian-Laird estimator for** ${\hat{\boldsymbol{\tau}}}^{\boldsymbol{2}}$ |
| --- | --- |
| **95% prediction interval** | -19.95 to 5.18 |
| **Expected proportion of studies** | Beneficial^#^ effect: 65.2%  Not important^#^ effect: 32.2%  Harmful effect^#^: 2.6% |
|  | **REML estimator for** ${\hat{\boldsymbol{\tau}}}^{\boldsymbol{2}}$ |
| **95% prediction interval** | -19.45 to 4.69 |
| **Expected proportion of studies** | Beneficial^#^ effect: 65.7%  Not important^#^ effect: 32.1%  Harmful effect^#^: 2.2% |
|  | **Paule-Mandel estimator for** ${\hat{\boldsymbol{\tau}}}^{\boldsymbol{2}}$ |
| **95% prediction interval** | -18.83 to 4.09 |
| **Expected proportion of studies** | Beneficial^#^ effect: 66.4%  Not important^#^ effect: 31.8%  Harmful effect^#^: 1.8% |

$\hat{\tau}^{2}$ represents the estimated between-study variance

# beneficial effect MD ≤ -5, not important effect MD > -5 to < 5, MD ≥ 5

MD: mean difference; REML: Restricted maximum-likelihood estimator
